# A Potential Role for Integrin-Linked Kinase in Colorectal Cancer Growth and Progression via Regulating Senescence and Immunity

**DOI:** 10.3389/fgene.2021.638558

**Published:** 2021-06-07

**Authors:** Saleh Almasabi, Afsar U. Ahmed, Richard Boyd, Bryan R. G. Williams

**Affiliations:** ^1^Centre for Cancer Research, Hudson Institute of Medical Research, Clayton, VIC, Australia; ^2^Cartherics, Hudson Institute of Medical Research, Clayton, VIC, Australia; ^3^Clinical Laboratory Sciences, Applied Medical Sciences, Najran University, Najran, Saudi Arabia; ^4^Department of Molecular and Translational Sciences, Faculty of Medicine Nursing and Health Sciences, Monash University, Clayton, VIC, Australia

**Keywords:** integrin-linked kinase, colorectal cancer, senescence, immunity, combination theraoy

## Abstract

Integrin-linked kinase (ILK) has been implicated as a molecular driver and mediator in both inflammation and tumorigenesis of the colon. ILK functions as an adaptor and mediator protein linking the extracellular matrix with downstream signaling pathways. ILK is broadly expressed in many human tissues and cells. It is also overexpressed in many cancers, including colorectal cancer (CRC). Inflammation, as evidenced by inflammatory bowel disease (IBD), is one of the highest risk factors for initiating CRC. This has led to the hypothesis that targeting ILK therapeutically could have potential in CRC, as it regulates different cellular processes associated with CRC development and progression as well as inflammation in the colon. A number of studies have indicated an ILK function in senescence, a cellular process that arrests the cell cycle while maintaining active metabolism and transcription. Senescent cells produce different secretions collectively known as the senescence-associated secretory phenotype (SASP). The SASP secretions influence infiltration of different immune cells, either positively for clearing senescent cells or negatively for promoting tumor growth, reflecting the dual role of senescence in cancer. However, a role for ILK in senescence and immunity in CRC remains to be determined. In this review, we discuss the possible role for ILK in senescence and immunity, paying particular attention to the relevance of ILK in CRC. We also examine how activating Toll-like receptors (TLRs) and their agonists in CRC could trigger immune responses against cancer, as a combination therapy with ILK inhibition.

## Introduction

The development of colorectal cancer (CRC) is a multistage process during which mutations in epithelial cells of the intestinal inner layer accumulate. In the early stages of CRC, benign polyps are formed; however, an accumulation of specific mutations in these polyps result in the formation of adenomas, which have a potential to develop to a more advanced stage of cancer. Subsequently, tumors can spread and metastasize to different areas of the body via the lymphatic system and blood vessels (Australian Institute of Health and Welfare, 2018).

According to the International Agency for Research on Cancer, in 2018 there were about 1.85 million new cases of CRC and 900,000 deaths in both sexes globally (International Agency for Research on Cancer, 2018). The CRC is the third most commonly diagnosed cancer and the second leading cause of cancer deaths worldwide (International Agency for Research on Cancer, 2018). CRC is the third most commonly diagnosed cancer in males and the second most commonly diagnosed cancer in females worldwide (Ferlay et al., 2019; Keum and Giovannucci, 2019). There are several different risk factors for CRC that include behavioral, environmental and biomedical risks. For example, alcohol use, diet, physical inactivity, tobacco use, occupation, diabetes, and obesity all contribute to the risk of CRC (Australian Institute of Health and Welfare, 2019). Inflammation, as evidenced by inflammatory bowel disease (IBD), is one of the highest risk factors for initiating CRC (Terzić et al., 2010). The 5 year relative survival rate is dependent on the stage of diagnosis and is influenced by the fact that this cancer is asymptomatic until later stages. For example, the survival rate at stage I is 100%, but this falls to only 13% at stage IV (Australian Institute of Health and Welfare, 2019).

The classification of CRC depends on etiology and genetics. There are three main groups: sporadic, familial and hereditary. The sporadic group represents the majority (75%) of CRC cases (Nojadeh et al., 2018). This group can be further divided into two subsets: microsatellite instability (MSI) and microsatellite stability (MSS), and they represent 15 and 85% of cases, respectively (Fernandes et al., 2018). MSI colorectal carcinoma is characterized by high immune cell infiltration (Smyrk et al., 2001; Llosa et al., 2015) and features a large number of mutations (Segal et al., 2008). This type is characterized by hot or inflamed tumors and it displays a better response to immunotherapy (Llosa et al., 2015).

Many studies have shown that tumor-infiltrating immune cells are associated with prognosis and metastasis of CRC. IBD is a chronic inflammation of the gastrointestinal tract (GIT) and is an important risk factor for developing and promoting colon cancer, i.e., colitis-associated cancer (CAC). IBD presents as two types: ulcerative colitis (UC) and Crohn’s disease (CD). CD affects the small intestine and large intestine, in addition to the mouth, esophagus, stomach and the anus, while UC mainly affects the colon and the rectum (Baumgart and Carding, 2007). Inflammation contributes to all stages of colon cancer tumorigenesis and progression (Terzić et al., 2010). The infiltrating inflammatory cells produce a variety of pro-inflammatory cytokines, chemokines, and growth factors to promote an immune response. However, excessive immune responses lead to tissue damage and tumor development (Sobczak et al., 2014; Li et al., 2017). Furthermore, parainflammation, which is a low level of inflammation that is intermediate between homeostasis and classical inflammation, is commonly widespread in different cancers, including CRC (Aran et al., 2016).

Cellular senescence was identified more than five decades ago as an arrest of the cell cycle in cultured fibroblasts associated with limited proliferative capacity (Hayflick and Moorhead, 1961). It is currently recognized as a process that is induced in cells when they become aged and/or when they are exposed to different types of stress, including DNA damage, telomere uncapping, oxidative stress, oncogene activation, lack of nutrients and growth factors, and others (Ben-Porath and Weinberg, 2005). The cells are reprogrammed to block their proliferation to prevent future cell generations from damage (Ingram Jane et al., 2010). However, they are still active in metabolism and transcription and are able to produce different chemokines, cytokines, growth factors, and matrix remodeling enzymes, which is termed senescence-associated secretory phenotype (SASP) (Lasry and Ben-Neriah, 2015). SASPs secreted by senescent cells recruit immune cells for the elimination of the senescent cells and the suppression of tumorigenesis (Wen et al., 2007; Burton and Stolzing, 2018). Senescence induction by therapy, therapy-induced senescence (TIS), has been utilized as an anti-cancer therapy (Lleonart et al., 2009). The main argument for senescence being an anti-cancer strategy is the loss of cellular proliferation (Schosserer et al., 2017). However, in the last two decades it has been recognized that senescence is more complex, and several reports indicate that a senescence inflammatory response (SIR), which is related to SASP, has a major effect on the tumor microenvironment (TME), suppressing anti-tumor immunity and stimulating neighboring cells growth (Lasry and Ben-Neriah, 2015; Pribluda et al., 2015).

Integrin-linked kinase (ILK) has been implicated as a molecular driver and mediator in both inflammation and tumorigenesis of the colon (Yan et al., 2014; Ahmed et al., 2017). ILK functions as an adaptor and mediator protein linking the extracellular matrix with downstream signaling pathways such as protein kinase B (PKB/Akt), glycogen synthase kinase 3β (GSK3β) and nuclear factor kappa B (NF-κB) (Delcommenne et al., 1998; Troussard et al., 2003; Ahmed et al., 2017). ILK regulates and mediates different cellular processes, including differentiation, proliferation, survival, apoptosis, cell adhesion, angiogenesis, migration, and invasion (Hannigan et al., 1996; Persad and Dedhar, 2003; Assi et al., 2008; Chan et al., 2011; Wani et al., 2011; Rooney et al., 2016; Lu et al., 2017). ILK is broadly expressed in many human tissues and cells (Hannigan and Dedhar, 1997). It is also overexpressed in many cancers, including colorectal cancer (CRC) (Bravou et al., 2006; Shen et al., 2016; Tsoumas et al., 2018). In addition, ILK has been shown to regulate inflammation which, as discussed above, is an important risk factor for developing CRC. ILK regulates inflammation in the mouse model of experimental colitis (Ahmed et al., 2017), and regulates growth and proliferation in colitis-associated cancer (CAC) (Assi et al., 2008). ILK is also involved in tumorigenesis (Chan et al., 2011) and cancer progression (Persad and Dedhar, 2003; Bravou et al., 2006).

More importantly, although there is less understanding of the role for ILK in cellular senescence in the cancer context, some reports that have suggested an involvement for ILK in cellular senescence regulation. One study has shown that cellular senescence is induced and suppressed skin tumors and benign colon adenomas by peroxisome proliferator-activated receptor-β/δ (PPARβ/δ) via repressing ILK expression and Akt phosphorylation (pAkt) (Zhu et al., 2014). Another study has found that inhibiting ILK in retinoblastoma and glioblastoma cell lines induced senescence markers (Duminuco et al., 2015). Nonetheless, there is less understanding about the role of ILK in senescence and its inflammatory response or SIR in a cancer context.

In this review, we aim to focus on a possible association of ILK with cellular senescence regulation and cancer immunity in CRC. This review summarizes up-to-date studies of ILK and its crosstalks with signaling pathways involved in cancer growth and progression. Also, we analyze the possible role of ILK in the inflammatory response of senescence which is considered the deleterious factor in tumor progression and cancer immunity. We also examine how activating Toll-like receptors (TLRs) could trigger anti-tumor immune responses and be used as a combination therapy with ILK inhibition in CRC.

### ILK Structure and Multi-Protein Binding

**FIGURE 1:**
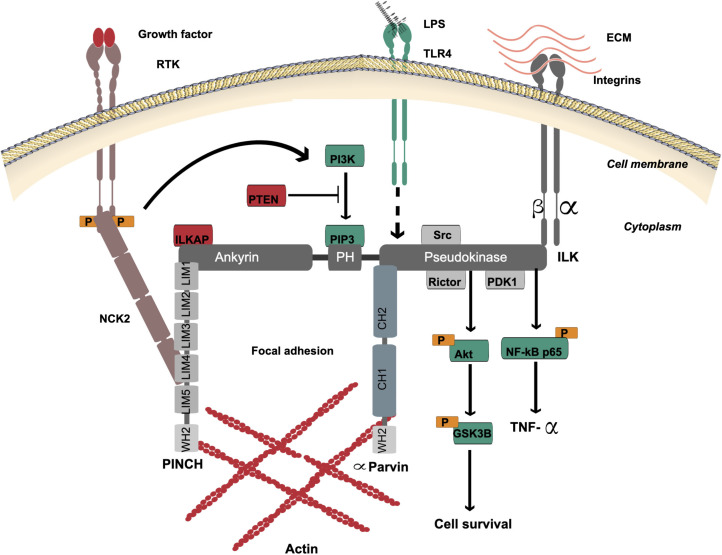
ILK structure and multi-protein binding. The localization of ILK is to focal adhesions. ILK contains three distinct domains: four repeats of amino-terminal ankyrin, a central PH-like domain and a carboxy-terminal pseudokinase catalytic domain. The ankyrin repeat domain binds the negative regulator of ILK, ILKAP, and also binds PINCH, which interacts with NCK2 for connection of ILK with growth factor receptors (RTK). The PH-like domain is in the center of ILK protein that binds PIP3, which is essential for activation of ILK via PI3K. PIP3 is dephosphorylated by tumor suppressor PTEN to negatively regulate the ILK activation. The ILK pseudokinase domain interacts with cytoplasmic domains of β1 and β3-integrin subunits and different actin-binding adaptor proteins such as α-Parvin. The interaction of ILK with PINCH and α-Parvin mediates the communication between the ECM and actin. The main ILK-binding proteins, which are involved in cell signaling via the pseudokinase domain, are PDK1, Rictor and Src. The ILK pseudokinase domain also interacts with and regulates the Akt-GSK3β signaling pathway. ILK mediates activation of the TLR4/NF-κB/TNF-α signaling pathway by lipopolysaccharides (LPS). The dotted line indicates the proposed mechanism of downstream activation.

ILK was first discovered in 1996 as an intracellular serine/threonine kinase and adaptor protein interacting with β1 integrin cytoplasmic domain (Hannigan et al., 1996). ILK is able to interact with cytoplasmic domains of β1 and β3-integrin subunits (Hannigan et al., 1996), and as predicted localizes to focal adhesions and myofilaments (Figure 1). While ILK phosphorylated serine and threonine residues of β1 integrin indicating its function as a protein kinase (Hannigan et al., 1996), it is now defined as a pseudokinase since its kinase-like domain lacks key active sites and likely functions as a non-catalytic signal transducer (Vaynberg et al., 2018). The gene that encodes ILK maps to the distal tip of human chromosome 11, at band 11p15.4/15.5 (Hannigan et al., 1997) and the single mRNA transcript encodes a protein of 452 amino acids that contains three distinct functional domains (Figure 1), including four repeats of an amino-terminal ankyrin domain, a central pleckstrin homology (PH)-like domain and a carboxy-terminal pseudokinase catalytic domain (Hannigan et al., 1997). The function of ILK as an adaptor and mediator protein links the extracellular matrix with downstream signaling pathways (Wu and Dedhar, 2001). In addition, it has shown that ILK localizes to centrosomes and regulates mitosis (Fielding et al., 2008). Centrosome clustering is regulated by ILK in cancer cells and this is an important process for cell survival (Fielding et al., 2011; Sikkema et al., 2014).

The ankyrin domain of ILK binds adaptor proteins such as particularly interesting new cysteine-histidine-rich protein (PINCH) (Tu et al., 1999) and ILK-associated phosphatase (ILKAP), a negative regulator of ILK (Leung-Hagesteijn et al., 2001). PINCH interacts with NCK2 to connect ILK with receptor tyrosine kinases (RTK) (Tu et al., 1998) including epidermal growth factor receptor (EGFR), fibroblast growth factor receptor (FGFR), vascular endothelial growth factor receptor (VEGFR), platelet derived growth factor receptor (PDGFR), and transforming growth factor-beta receptor (TGFβR) (Shi and Chen, 2017; Urner et al., 2019).

The PH-like domain is located in the center of the ILK protein and binds phosphatidylinositol 3,4,5-triphosphate (PIP3), which is essential for activation of ILK via phosphoinositide 3-kinase (PI3K) (Delcommenne et al., 1998). PIP3 is dephosphorylated to phosphatidylinositol 4,5-diphosphate (PIP2) by tumor suppressor PTEN to negatively regulate ILK activation (Obara et al., 2004). PTEN deficiency in cells leads to high PIP3 levels and constant activation of ILK and PKB/Akt (Persad et al., 2000).

The ILK pseudokinase domain interacts with cytoplasmic domains of β1 and β3-integrin subunits and different actin-binding adaptor proteins, including α-Parvin and Paxillin (Hannigan et al., 1996; Wu, 2004; Rooney et al., 2016). The main ILK-binding proteins, which are involved in cell signaling via the pseudokinase domain, are PDK1, Rictor and Src (Delcommenne et al., 1998; Kim et al., 2008; McDonald et al., 2008b). Rictor colocalization with ILK via this domain is necessary for Akt phosphorylation (McDonald et al., 2008b). Also, ILK and Rictor form a complex to regulate TGF-β function (Serrano et al., 2012). ILK pseudokinase domain interacts with and regulates the Akt-GSK3β signaling pathway (Delcommenne et al., 1998; Pap and Cooper, 1998; Troussard et al., 2003). The phosphorylation of GSK3 inhibits its activity and this can be mediated by ILK overexpression (Delcommenne et al., 1998).

The dynamic communication between the extracellular matrix (ECM) and actin is mediated by ILK in its role as an adaptor and mediator protein to support cell adhesion, spread and migration. This is mediated when integrin binds to the ILK/PINCH/Parvin complex, termed IPP (Wickström et al., 2010). A tight complex is formed to localize to focal adhesion (Zhang et al., 2002a; Wu, 2004). There are two isoforms of PINCH containing five LIM domains and the ILK ankyrin domain binds LIM1 (Yang et al., 2009). Also, there are three isoforms of Parvin, and all contain two CH domains; the ILK pseudokinase domain binds to the CH2 domain of α-Parvin (Fukuda et al., 2009). A recent study by Vaynberg et al. found that WASP-Homology-2 (WH2) actin-binding motifs in both PINCH and α-Parvin organize actin bundling, which is triggered by Mg-ATP binding to ILK (Vaynberg et al., 2018). Blocking the ATP-binding site in the ILK pseudokinase domain disrupted actin bundling (Vaynberg et al., 2018).

ILK mediates activation of Toll-like receptor 4 (TLR4)/NF-κB/tumor necrosis factor alpha (TNF-α) signaling pathway by lipopolysaccharide (LPS), a major component of the outer membrane of Gram-negative bacteria such as *Helicobacter pylori* (Ahmed et al., 2014). Since *H. pylori* activates both EGFR and TLRs, and blocking EGFR signaling inhibits TLR activation of downstream signaling pathways (Chattopadhyay et al., 2015), it remains possible that ILK is a mediator of both growth factor and TLR activities.

### The Role of ILK in Different Contexts

#### The Role of ILK in Embryo and Normal Contexts

ILK is broadly expressed in many human tissues and cells (Hannigan and Dedhar, 1997), where it is implicated in the regulation of different cellular processes depending on context, including differentiation, proliferation, survival, apoptosis, cell adhesion, angiogenesis, migration, and invasion (Hannigan et al., 1996; Persad and Dedhar, 2003; Assi et al., 2008; Chan et al., 2011; Wani et al., 2011; Rooney et al., 2016; Lu et al., 2017). ILK is necessary for embryonic development (McDonald et al., 2008a), as its ablation in embryonic models (*Xenopus laevis*, mice and zebrafish) is lethal, affecting adhesion and migration processes as well as vasculature development (Yasunaga et al., 2005; Bendig et al., 2006; Moik et al., 2013).

In normal contexts, ILK has a variety of functions that are dependent on cell or tissue types. One study showed that ILK deletion in mammary glands of mice and in 3D culture leads to the failure of mammary epithelial cells to form polarized acini, due to destabilization of microtubules (Akhtar and Streuli, 2013). Mutations in ILK that result in the interruption of binding with its partner, Parvin, prevent the differentiation of mammary epithelial cells in response to prolactin, a hormone that acts on mammary glands during pregnancy (Rooney et al., 2016). The inhibition of interaction between ILK and PINCH in hamster ovary epithelial cells delays the change in cell shape after plating, which in turn impairs cellular motility (Zhang et al., 2002b). Similarly in skin, ILK deletion in keratinocytes leads to defects in adhesion, spread and migration of cells, as well as a defect in basement membrane integrity *in vivo* and *in vitro* (Lorenz et al., 2007). In this study, ILK ablation does not affect proliferation, but rather alters the location of proliferating epidermal cells (Lorenz et al., 2007), whereas another study showed that proliferation of keratinocytes is impaired (Nakrieko et al., 2008). Mouse hepatocytes lacking ILK exhibit decreased matrix-induced differentiation (Gkretsi et al., 2007a), and apoptosis is induced without affecting Akt phosphorylation (Gkretsi et al., 2007b).

More specifically, in the context of normal intestinal epithelium, at early time points of seeding of normal human intestinal epithelial cells (after 4 h), ILK knockdown (KD) does not affect the adhesion rate. However, it reduces the spread of cells, as they remained rounded for a longer time (∼18 h) in comparison with ILK wild-type (WT) cells (Gagne et al., 2010). In addition to the cell adhesion and spread, ILK KD cells also experience less migratory and proliferative activity (Gagne et al., 2010). ILK-associated c-Src mediates a dynamic actin polymerization by interacting with and phosphorylating cofilin during adhesion of normal rat intestinal epithelial cells, but not suspended cells (Kim et al., 2008).

#### The Role of ILK in Non-cancer Diseases

ILK has been revealed to contribute to different non-cancer diseases. The most important related to cancer development is inflammation. ILK KO in epithelial cells of the mouse intestine displays a reduction in inflammation of the colon (colitis) and inflammation-induced cancer (CAC) (Assi et al., 2008, Assi et al., 2011b). Moreover, previous studies from our laboratory have shown that myeloid-ILK deficiency reduced intestinal inflammation in experimental colitis by regulating neutrophil infiltration and cytokine production (Ahmed et al., 2017). ILK is also required for mediating LPS-induced inflammatory gene expression in endothelial cells (Hortelano et al., 2010). ILK inhibition in endothelial cells reduces leucocyte adhesion and migration in Trans-endothelial migration assays (Hortelano et al., 2010). Also, ILK deletion in a mouse model prevents angiotensin II-induced inflammation via reducing macrophages and lymphocytes infiltration as well as proinflammatory secretions of the kidney (Alique et al., 2014). These studies suggest that ILK plays an essential role in different cell types mediating inflammatory induction.

Furthermore, ILK contributes to other diseases. For example, ILK inhibition in polycystic kidney disease in mouse models has shown a reduction in fibrosis cyst growth with improved renal function and survival (Raman et al., 2017). ILK also plays a role in cardiac hypertrophy as indicated by that high expression in human cardiac hypertrophic ventricles (Lu et al., 2006). ILK inhibition attenuated the induced hypertrophy (Lu et al., 2006). In contrast, ILK overexpression has been implicated in myocardial infraction, a disease that leads to heart failure resulted from myocardial cell death (Zhang and Guo, 2018). Transplanting cardiac stem cells overexpressing ILK restores cell death and cardiac function in a mouse model of this disease (Zhang and Guo, 2018). Furthermore, the mouse Alzheimer’s disease model displays a decrease in ILK expression, whereas its overexpression rescues hippocampal neurogenesis and defects in memory via the downstream signaling pathway, Akt/GSK3β (Xu X.F. et al., 2018).

#### The Role of ILK in Cancer

ILK has been proposed to play a critical role in cancers. It has been established for about two decades that ILK overexpression and dysregulation are associated with development and progression of different cancers, including CRC (Marotta et al., 2003; Hannigan et al., 2005; Koul et al., 2005; Bravou et al., 2006; Edwards et al., 2008; Assi and Salh, 2011; Assi et al., 2011a; Matsui et al., 2012; Sikkema et al., 2014; Yan et al., 2014; Tsoumas et al., 2018). ILK is implicated in tumorigenesis (Zheng et al., 2019), since its knockdown in hepatocellular carcinoma cells as well as ovarian cancer cells impairs tumor growth in mouse model (Chan et al., 2011; Li et al., 2013). Also, its overexpression is associated with a poor survival rate of cancer patients (Graff et al., 2001; Ahmed et al., 2003; Dai et al., 2003). For instance, the expression of ILK in human tissues from primary colorectal carcinomas and lymph node metastases examined by immunohistochemistry (IHC) reveal expression levels as very low, high and significantly higher in normal tissue, primary tumor and lymph node metastases, respectively (Bravou et al., 2003, 2006).

ILK is involved in regulation of epithelial-mesenchymal transition (EMT), an important cellular process regulating migration, invasion and chemoresistance in CRC (Bravou et al., 2006; Assi et al., 2008; Yan et al., 2014; Tsoumas et al., 2018). Artificial overexpression of ILK in the SW480 CRC cell line elevates EMT-related proteins, as well as inducing invasive and migratory activities (Yan et al., 2014). This is in accord with IHC examination of human colorectal carcinoma tissues, which showed that the expression of EMT-related proteins was positively correlated with ILK expression (Bravou et al., 2006). Chemoresistant CRC cell lines showed a sensitivity to chemotherapy and downregulation of EMT markers following ILK inhibition (Tsoumas et al., 2018).

Furthermore, ILK overexpression has a central role in apoptotic resistance. ILK knockout in HT29 CRC cells under hypoxia conditions induces apoptosis, as well as decreases invasive activity (Xiao et al., 2014). In gastric carcinoma SGC7901/DDP cells, overexpression of ILK induces multidrug resistance mediated by Akt phosphorylation (Song et al., 2012).

Therefore, while the function of ILK is diverse in different contexts, nonetheless, based on the above studies, ILK is implicated in normal and disease contexts via utilizing its critical function to mediate or regulate several cellular processes including cell shape, adhesion and tissue integrity, as well as cell growth, invasion and migration. Such cellular processes are regulated by dynamic actin organization which is well known to be regulated by ILK. In addition, ILK is implicated as an important molecule in different compartments such as epithelial, endothelial and inflammatory cells to mediate cellular processes regulation and inflammatory response.

### ILK Mediates Regulation of Different Cellular Processes in CRC via Cross-Talk With Different Cell Signaling Pathways

**FIGURE 2:**
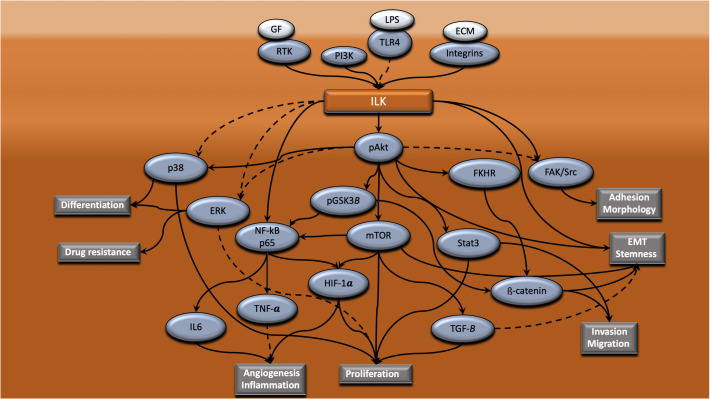
ILK mediates regulation of different cellular processes in CRC via cross-talk with different intracellular signaling pathways. ILK is a central mediator of signaling cascades that regulate a range of cellular processes that are vital to the progression of CRC. ILK coordinates the connection of integrins, RTK and TLR4 with downstream signaling pathways, resulting in the regulation of processes such as adhesion, morphology, EMT, stemness, invasion, migration, proliferation, angiogenesis, inflammation, drug resistance and differentiation. Dotted lines indicate that this is yet to be shown in CRC.

ILK interacts with different critical signaling pathways (Zheng et al., 2019) regulating important cellular processes that have an implication in inflammation and cancer. The signaling pathways that interact with ILK in the CRC context are summarized in Figure 2. From this, it is obvious that upregulation of ILK is associated with dysregulation of the PI3K-Akt-GSK3β signaling pathway as revealed in human CRC tissues (Zhang et al., 2019), and that pathway is well known to be mediated via ILK (Figure 1; Delcommenne et al., 1998). But other pathways are also involved.

#### Epithelial-Mesenchymal Transition (EMT)

The EMT cellular process in cancer is regulated by diverse signaling pathways that have been implicated as being regulated either directly by ILK or indirectly via ILK downstream signaling pathways. For instance, ILK overexpression in human CRC is associated with high expression of EMT markers, including ZEB, Snail, β-catenin and low expression of E-cadherin (Tsoumas et al., 2018). Similarly, *in vitro* ILK upregulation in SW480 colorectal cancer cells promotes upregulation of Snail, slug, vimentin and MMP9, subsequently increasing proliferative, migratory and invasive activities (Assi et al., 2008; Yan et al., 2014; Shen et al., 2016). Furthermore, activation of the Akt-FKHR pathway is correlated with ILK overexpression in primary human CRC tissues and is associated with nuclear β-catenin expression and downregulation of E-cadherin, indicating EMT induction (Bravou et al., 2006). Also, ILK inhibition in colon cancer cells reduces tumor growth in mouse models and *in vitro* cell lines via reducing β-catenin nuclear expression and increasing GSK3β activity by phosphorylation reduction, resulting in β-catenin degradation (Tan et al., 2001). Snail expression is also suppressed whereas E-cadherin expression is induced by ILK inhibition (Tan et al., 2001). Additionally, the PI3K/Akt/mammalian target of rapamycin (mTOR) pathway is highly activated in colon cancer stem cells and inhibiting this pathway reduces stem cell proliferation or spheroid formation; the stemness is reduced as indicated by decreasing expression of Lgr5, a cancer stem cell marker (Chen et al., 2015). This marker, as well as ZEB, was found to be colocalized with ILK in human CRC tissues by immunofluorescence (Tsoumas et al., 2018). Furthermore, in breast cancer cells, an ILK-Rictor complex regulates TGF-β function for EMT induction and this complex is absent in normal cells (Serrano et al., 2012). Whether this plays a role in CRC or is cell type- or tissue context-dependent has yet to be determined. A microRNA, MiR-542-3p, is downregulated in human CRC cancers and introducing this microRNA into HCT116 and SW620 CRC cells changes the morphology of the cells and reduces cell adhesion, as well as suppressing invasion via downregulation of the ILK/FAK/c-Src pathway (Oneyama et al., 2012). Therefore, ILK interacts directly or indirectly with different proteins to regulate stemness and EMT, implicating a role for ILK in cancer progression and metastasis.

#### Angiogenesis and Inflammation

Tumor angiogenesis and inflammation are important factors in cancer progression. Although there are few studies in colon cancer showing direct angiogenesis induction by ILK, one report has revealed that ILK overexpression regulates mesenchymal stem cell survival and angiogenesis via Akt and mTOR phosphorylation and VEGF expression (Zeng et al., 2017). Another study showed that ILK overexpression in melanoma cells promotes angiogenesis of endothelial cells by activating the NF-κB/IL-6 signaling pathway *in vitro* and *in vivo* (Wani et al., 2011). NF-κB and IL-6 and other inflammatory cytokines are also induced by ILK in experimental colitis and colorectal cancer (Yan et al., 2014; Ahmed et al., 2017). Furthermore, a study has reported that the Akt-mTOR signaling pathway which is known to be regulated by ILK (Zeng et al., 2017) is also involved in angiogenesis in colon cancer. For example, Cryptotanshinone, which is derived from *Salvia miltiorrhiza Bunge*, suppresses CT26 colon cancer cell angiogenesis via inhibiting the PI3K/Akt/mTOR signaling pathway, nuclear hypoxia inducible factor-1α (HIF-1α), VEGF and VEGFR gene activation (Zhang et al., 2018). Different inflammatory proteins are also suppressed in colon cancer tissues, including interleukin-4 (IL-4), IL-6, IL-18, TNF-α, and interferon gamma (IFN-γ) (Zhang et al., 2018).

As discussed above, inflammation is one of the highest risk factors for CRC. ILK has a key role in inflammation by regulating the production of TNF-α via NF-κB activation (Ahmed et al., 2014, 2017; Yan et al., 2014; Krenn et al., 2016). The cell wall of H. pylori, which is highly associated with chronic inflammation and gastric cancers, contains LPS, which activates TLR4 and the downstream NF-κB signaling pathway, thereby inducing the production of pro-inflammatory mediators including TNF-α, IL-1β, IL-8, and others (Sandborn and Hanauer, 1999; Pålsson-Mcdermott and O’Neill, 2004). Our laboratory has found that LPS induces ILK-dependent phosphorylation of Akt and GSK3β, as well as phosphorylation of NF-κB p65 at Ser536 and TNF-α production in gastric cancer cells (Ahmed et al., 2014). ILK inhibition blocks the LPS-induced phosphorylation of Akt, GSK3β, and NF-κB p65 phosphorylation, as well as TNF-α production (Ahmed et al., 2014). A similar study showed that ILK mediates LPS activation and is required for inflammatory induction in endothelial cells (Hortelano et al., 2010). This study found that ILK inhibition in these cells reduced LPS-induced adhesion molecules, ICAM-1 and VCAM-1. This subsequently decreased the adhesion and trans-endothelial migration of monocytes and lymphocytes *in vitro* (Hortelano et al., 2010).

The transcription factor Signal transducer and activator of transcription 3 (STAT3) may be regulated by ILK or Akt in inflammatory and cancer contexts. Our laboratory has shown that myeloid-ILK-deficient cells in an experimental colitis model exhibited reduced activation of NF-κB and PI3K signaling pathway, but elevated STAT3 activation and proliferation of intestinal epithelium (Ahmed et al., 2017). Consequently, the production of inflammatory cytokines was suppressed, including TNF-α, IL-6, and IL-1β (Ahmed et al., 2017). In contrast, ILK may regulate STAT3 indirectly via its dependent pathway, PI3K/Akt, in cancer. In SW480 CRC cells, overexpression of miR-199a decreased the expression of pPI3K, pAkt, p-JAK1, and p-STAT3, which subsequently suppressed proliferation, migration and invasion, but induced apoptosis (Zhu et al., 2018). Thus, these two unrelated observations in different cellular systems suggest that ILK may indirectly regulate Stat3 in a context-dependent manner.

#### Cell Death

Mitochondrial dysfunction has an important role in cancer progression and resistance to apoptosis by altering metabolism (Hsu et al., 2016). Hypoxia induces the expression of ILK along with HIF-1α in CRC HT29 cells and induces survival and invasion (Xiao et al., 2014). Interestingly, mitochondrial integrity is protected from apoptosis in this condition via ILK. In contrast, ILK knockout (KO) under hypoxic conditions induces apoptosis by decreasing Bcl-2 expression and increasing Caspase-3 activity, besides suppressing proliferation and invasion (Xiao et al., 2014). This suggests that ILK could have an influence on mitochondrial function in CRC. Human CRC tissues have high levels of glucose transporter 1 (Glut1), which regulates energy metabolism, and in CRC HCT116 cells Glut1 induces proliferation and apoptotic resistance via activation of PI3K, Akt, mTOR, TGF-β1, and Bcl-2 expression, and inactivation of PTEN, Bax, cleaved caspase-3, and cleaved PARP (Wu et al., 2018). Silencing Glut1 in CRC HCT116 cells inhibited the activation of these signaling pathways and their functional consequences (Wu et al., 2018). A more recent study has shown that the Akt inhibitor SC66 induced apoptosis in CRC via the Akt/GSK3β/Bax axis *in vitro* and *in vivo* (Liu et al., 2019).

Although there are insufficient studies linking ILK and MAPK (p38, Erk1/2, and JNK) signaling in colon cancer, some studies have demonstrated such an interaction in different cell types. For example, in gastric cancer cells, ILK silencing inhibited drug resistance via pAkt and pErk suppression (Song et al., 2012). Furthermore, a study in osteosarcoma cells found that ILK inhibition led to suppression of p38 and Erk1/2 but not JNK, and this was implicated in the cellular proliferation and differentiation (Wang et al., 2014). In addition to these studies, in a CRC context, suppression of pAkt induces cell death in SW620 colon cancer cells via activation of MAPK p38, and that in turn mediates the expression of senescent marker p21 and autophagic marker LC3 (Nagappan et al., 2017). Moreover, Twist-induced EMT in breast cancer cells is associated with upregulation of ILK, FAK, PI3K/Akt, and Erk, but downregulation of p53 (Yang et al., 2016).

Therefore, ILK has numerous interactions with different cell signaling pathways and also mediates communications among these pathways, indicating its unique function and potential to be a therapeutic target and modulate the TME and suppress tumor growth.

#### Tumor Microenvironment (TME)

**FIGURE 3:**
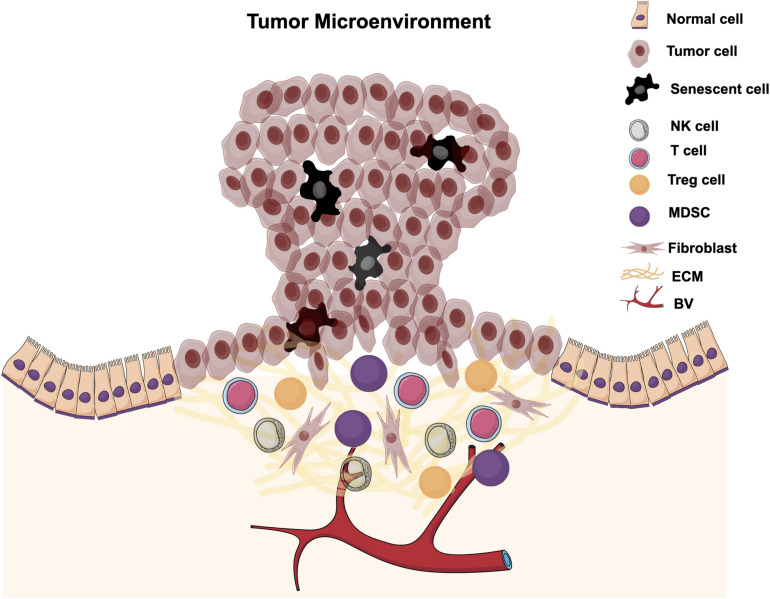
Tumor microenvironment. The tumor microenvironment is the local area surrounding a tumor and is infiltrated with different cellular components and factors. TMEs are induced and modified by interaction with tumor cells to promote tumor progression and metastasis.

The tumor microenvironment is an area surrounding a tumor that has different cellular components and factors that support tumor growth and progression (Figure 3). TME components include immune cells, fibroblasts, induced senescent cells, vasculatures, soluble factors, and ECM. TMEs are induced and modified by interaction with tumor cells to favor tumor progression and metastasis. The interaction between tumor cells and different TME components can be either direct via cell-cell interaction or autocrine via interacting with different cytokines, chemokines, growth factors, and enzymes. Different factors in the TME, such as induced senescent cells and their SASP secretions and recruited immune cells are implicated in cancer growth and progression via different cellular and molecular mechanisms. These factors will be discussed in the following sections and a possible role for ILK in these factors’ regulation.

### Cellular Senescence and Its Contribution to Cancer Progression and Therapy

#### Cellular Senescence

Senescence was identified more than five decades ago as an arrest of the cell cycle in cultured fibroblasts due to limited proliferative capacity (Hayflick and Moorhead, 1961). It is currently recognized as a process that is induced in cells when they become aged and/or when they are exposed to different types of stress, including DNA damage, telomere uncapping, oxidative stress, oncogene activation, lack of nutrients and growth factors, and others (Ben-Porath and Weinberg, 2005). The cells are reprogrammed to block their proliferation to prevent future cell generations from damage (Ingram Jane et al., 2010). However, they are still active in metabolism and transcription and they are able to produce different chemokines, cytokines, growth factors, and matrix remodeling enzymes, which is termed SASP (Lasry and Ben-Neriah, 2015).

There are different morphological and molecular phenotypes for induced senescent cells. Microscopically, senescent cells exhibit a size enlargement and flattened shape (Chien et al., 2011; Frey et al., 2018). The nucleus is prominent and enlarged and displays senescence-associated heterochromatin foci (SAHF) by DAPI staining under a microscope (Chien et al., 2011; Frey et al., 2018). High activity of senescence associated-beta galactosidase (SA-β-gal) is a characteristic of senescent cells at pH 6 using specific histochemical staining (Duminuco et al., 2015). Molecularly, senescent cells show an upregulation of tumor suppressor gene p53 and cyclin-dependent kinase suppressors such as p21 and p16 (Chien et al., 2011; Frey et al., 2018). The proliferative marker Ki67 is absent in senescent cells (Xue et al., 2011). They also upregulate negative regulators of apoptosis such as Bcl 2 (Saleh et al., 2020b).

#### Dual Role of Senescence in Cancer

In early studies, senescence was thought to have an anti-tumor impact. Besides arresting cell growth, SASPs secreted by senescent cells recruit immune cells eliminating the senescent cells suppressing tumorigenesis (Wen et al., 2007; Burton and Stolzing, 2018). This cellular process is induced in preclinical cancer models including CRC by numerous drugs and is referred to as TIS (Bojko et al., 2019; Saleh et al., 2020a). This type of senescence induction has been considered as an anti-cancer therapy (Lleonart et al., 2009). The main arguments for senescence being anti-cancer is loss of cellular proliferation (Schosserer et al., 2017).

**FIGURE 4:**
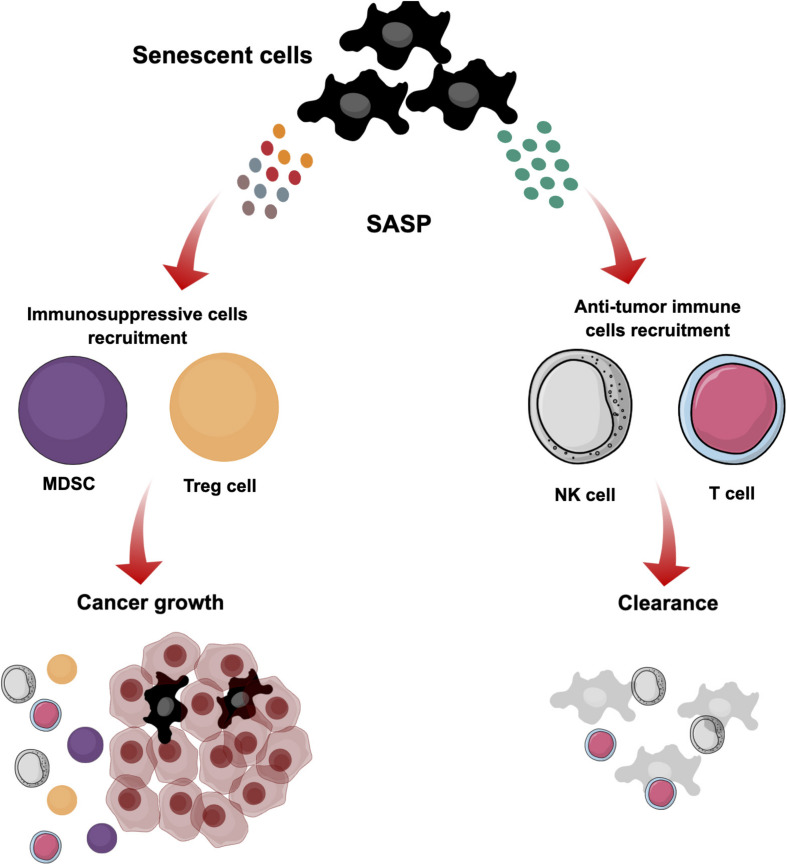
The dual role of cellular senescence. Senescent cells are induced in the TME by different stimuli. These cells stop proliferating but maintain active metabolism and transcription. They produce different SASP secretions that reflect the dual role of senescence. They can produce particular SASP secretions to recruit immune cells such as NK and T cells for clearance and suppression of tumorigenesis. However, modification of SASP secretions blocks the elimination of senescent cells by immune cells, and subsequently they can have a negative influence in the tissue microenvironment by supporting cancer growth and recruiting immunosuppressive cells such as MDSCs and Treg cells.

However, during last two decades it has been recognized that hallmarks of senescence are more complex and not only involve growth arrest as described above but also have an ability to drive cancer development and progression (Krtolica et al., 2001; Coppé et al., 2008; Schosserer et al., 2017; Hernandez-Segura et al., 2018). Senescent cells that promote cancer progression and recurrence are found in the tumor invasive front (Demaria et al., 2017; Young Hwa et al., 2017). This reflects a dual function of senescence in cancer (Figure 4). The function of senescence depends on context, genetic background and duration of accumulation (Wen et al., 2007; Krizhanovsky et al., 2008; Eggert et al., 2016; Burton and Stolzing, 2018). SASP as a critical factor in senescence can be modified, this blocks immune cell-mediated elimination of senescent cells, and subsequently has a negative influence in the tissue microenvironment by rescuing cancer growth (Toso et al., 2014; Greten and Eggert, 2017; Schosserer et al., 2017). Therefore, senescence is complex and failure of immune cells to eliminate senescent cells after induction will promote cancer growth and progression.

Several reports have revealed that a subset of senescent cancer cells can escape from senescence (Wang et al., 2011, 2013; Saleh et al., 2020a). For instance, therapy-induced senescent H1299 non-small cell lung carcinoma cells have been captured by time-lapse live-cell imaging and by a senescence colony assay showing an escape from senescence and a return to replication (Wang et al., 2011, 2013). Similarly, CRC HCT116 cells were treated with doxorubicin until senescence was induced and SASP VEGF and IL8 were upregulated (Was et al., 2017). Doxorubicin was then removed and the HCT116 cells exhibited an escape from cell senescence and resumption of proliferation (Was et al., 2017). Therefore, the accumulation of senescent cells over time will affect the TME and will exhibit an escape from senescence or growth arrest. It has been shown that there are numerous senolytic drugs eliminating senescent cells that might be beneficial to prevent the harmful effects of senescence accumulation.

Senolytic drugs as adjuvant therapy in cancer are a promising approach to prevent deleterious senescence. These drugs selectively kill senescent non-proliferating cells. There are senolytic drugs that have been approved by FDA for purposes other than senolysis (Saleh et al., 2020a). Senolytics such as dastinib + quercetin and fisetin have shown a delay cancer progression and also death from age-related diseases in mouse models (Xu M. et al., 2018; Yousefzadeh et al., 2018; Kirkland and Tchkonia, 2020). The use of the senolytic drug ABT-263 following traditional senescent-inducing therapy improved tumor suppression for an extended period in a mouse model (Saleh et al., 2020b). However, there are possible challenges. For example, doxorubicin-induced senescent CRC HCT116 cells treated with bafilomycin A1, an autophagic inhibitor, showed a delay in the growth for a few days, but cell growth recovered (Was et al., 2017). The autophagic modulator has been thought to have a senolytic effect (Fuhrmann-Stroissnigg et al., 2017). In this study, the induced senescent HCT116 cells treated with bafilomycin A1 were implanted into NOD/SCID mice and exhibited greater tumor growth compared with doxorubicin-induced senescent HCT116 cells (Was et al., 2017). VEGF, an important SASP component, was found to be upregulated *in vitro* and *in vivo*, and could be responsible for cells escaping from senescence and supporting tumor growth (Was et al., 2017). Therefore, senolytic drugs might be beneficial but SASPs and signaling pathways involved in SASPs regulation must also be taken in consideration.

#### The Heterogeneity of SASPs and Their Derivation

The SASP secretions in senescence have been studied intensively. They are derived from stromal cells and there are reports that showed senescent epithelial cancer cells can also secrete SASPs (Coppé et al., 2008; Bojko et al., 2019; Saleh et al., 2020a; Wang et al., 2020). SASPs are secreted by different cell types including fibroblasts, vasculature smooth muscle cells, bone, and also from tumor-associated but unidentified cell types (Saleh et al., 2020a; Wang et al., 2020). Epithelial-derived cancer cells also can produce SASPs after senescence induction. For example, HCT116 CRC cells induced with doxorubicin secrete IL8 and VEGF (Was et al., 2017). Whereas 1205Lu melanoma cells induced with Palbociclib secrete IL6, IL8, and CXCL1 (Yoshida et al., 2016). Additionally, HepG2 liver cancer cells induced with 5-aza-2′-deoxycytidine secrete ICAM-1, IL1ra, and IL8 (Venturelli et al., 2013). Also, different cancer cell lines including HCT116 CRC, MDA-MB-231 breast cancer and A549 lung carcinoma cells treated with different chemotherapeutic agents secrete IL8 and VEGF (Bojko et al., 2019). MCF-7 breast cancer and SH-SY-5Y neuroblastoma cells secrete VEGF but did not secrete IL-8 (Bojko et al., 2019). The chemotherapeutic agents that induced senescence in these cell lines include doxorubicin, irinotecan, oxaliplatin, methotrexate, 5-fluorouracil and paclitaxel (Bojko et al., 2019). Also, ovarian cancer cell lines including OV1369 (R2), OV90, OV4453, and OV1946 induced with Olaparib secrete IL6 and IL8 (Fleury et al., 2019). The SASPs secreted from senescent cancer cells mentioned in these studies are inflammatory mediators suggesting that senescent cancer cells mimic inflammatory cells in their signaling and impact on tumor growth and progression (Lasry et al., 2017).

The SASP is heterogeneous and there are dominant pro-inflammatory components. The SIR or different distinct inflammatory molecules which partially related to SASP can switch the role toward tumor promotion (Pribluda et al., 2015; Kim et al., 2016). Targeting the SIR by anti-inflammatory agents affects CRC tumor growth (Pribluda et al., 2015). It has been reported that NF-κB p65 is activated in senescent cells and is considered as a master control of SASP in CRC as well as in many cancers (Chien et al., 2011; Pazolli et al., 2012; Bernal et al., 2014; Strzeszewska et al., 2018). The NF-κB activates SASPs and is involved in chemoresistance (Musiani et al., 2020). The NF-κB signaling pathway cross-talks with IL-6 and IL-1β activity in the intestinal epithelium (Liu et al., 2003; Wang et al., 2003). Such pro-inflammatory cytokines are implicated in both inflammation and EMT in the TME (Coppé et al., 2008, 2010; Pribluda et al., 2015; Eggert et al., 2016). Thus, inflammatory molecules secreted by senescent cells are likely involved in the complexity of senescence. These inflammatory molecules or mediators mimic secretions in TME and this suggests that targeting inflammatory response could prevent the harmful effects of senescence.

Signaling pathways that are implicated in inflammation and tumors can be targeted to modulate inflammatory response in senescence. For example, a study in *Pten*-null senescent prostate tumors has shown that SASP components can be reprogrammed by targeting Stat3 (Toso et al., 2014). A range of secretions were inhibited, including CXCL2, G-CSF, GM-CSF, M-CSF, C5a, IL-6, IL-10, and IL-13, whereas some secretions remained high, such as MCP-1 (CCL2), BCL, ICAM-1, CXCL10 (Toso et al., 2014). Subsequently, this influenced infiltration of different immune cells, including myeloid-derived suppressor cells (MDSCs), T cells and natural killer cells (NK cells), and induced an anti-tumor response (Toso et al., 2014). However, the nature of all secretory molecules associated with SASPs is complex and yet to be revealed, and this is essential in order to decipher their roles in the recruitment of immune cells to the TME.

### A Role for Senescence in Immunosuppressive TME via SASP Secretions

#### Immune Microenvironment

As there is a strong link between senescence and immunity (Coppé et al., 2010; Eggert et al., 2016), it is essential to understand immune cells and their functions in CRC and other solid tumors. The immune microenvironment comprises different cells derived from both the innate and adaptive immune systems (Grivennikov et al., 2010). The innate immune cells include tumor-associated macrophages (TAMs), MDSCs, mast cells (MCs), dendritic cells (DCs), neutrophils, and NK cells (Grivennikov et al., 2010). The adaptive immune cells are T cells and B cell subsets (Quante et al., 2013). The immune cells that are affected in the TME that mediate immune evasion are mainly T regulatory cells (Treg), MDSCs and TAMs or M2 macrophage. A high number of Treg cells in colorectal cancer is correlated with poorer prognosis (Takuro et al., 2016). The high level of expression of Treg cells is associated with suppression of the anti-CRC tumor immune response driven by CD4 T cells (Betts et al., 2012). Similarly, colonic MDSCs suppress cytotoxic CD8 T cell killing of colonic epithelial tumor cells (Katoh et al., 2013). Also, M2 macrophages promote growth and progression in CRC cells and is correlated with poor prognosis in CRC patients (Liu et al., 2020).

#### Senescence Mediating Immune Evasion

A role for senescence in immune evasion has recently been uncovered (Muñoz et al., 2019). Senescent cells express high NKG2D ligands such as MICA and MICB and these molecules can be recognized by the NKG2D receptor of NK cells to mediate cytotoxicity independent of p53 and p16 expression. However, a subset of senescent cells are still able to avoid immune recognition via shedding NKG2D ligands from their surfaces (Muñoz et al., 2019). MMPs were upregulated in the persistent senescent cells and involved in shedding the NKG2D ligands. Targeting the MMPs significantly enhanced NK cells killing against senescent cells (Muñoz et al., 2019). MMP is a SASP component that is highly secreted by senescent cells (Xu et al., 2019). Moreover, cellular senescence induced by chemotherapeutic agents stimulates PD-L1-mediating immune evasion (Xu et al., 2019). Normal stromal prostate cells treated with chemotherapeutic agents became senescent and upregulate senescence markers and secrete SASP molecules including AREG (Xu et al., 2019). This stromal molecule induced proliferation, invasion, and migration in prostate cancer cells. In addition, AREG stimulated PD-L1 expression in recipient prostate cancer cells (Xu et al., 2019). This provides evidence for a negative role for senescence secretions in immunosuppressive TME.

Different secretions of cytokines and chemokines in the TME also affect anti-tumor immunity. Sporadic CRC exhibits high CXCR2 secretion, which has an important role in induction of chronic inflammation and CAC development (Katoh et al., 2013). This chemokine receptor recruits MDSCs, which in turn suppress T cell functions (Katoh et al., 2013). Moreover, CCL2 expression contributes to progression of CRC and accumulation of MDSCs (Chun et al., 2015). This chemokine ligand influences the expression of reactive oxygen species (ROS) and inducible nitric oxide synthase (iNOS) in MDSCs accumulating in colorectal tumors and mediates T cell inactivation via T-cell receptor complex modifications (Chun et al., 2015). Moreover, CCL2 secreted from oncogene-induced senescent cells, another type of senescence induction mediated by oncogene activation, stimulates MDSCs and inhibits NK cells and promotes liver tumor growth (Eggert et al., 2016). Although CCL2 in the above studies displayed an immunosuppressive function, another study in prostate cancer found that the immunosuppression was due to different additional secretions accompanied with CCL2 secreted by senescent cells and modulation of these secretions activated the anti-tumor immunity while maintaining high CCL2 levels (Toso et al., 2014). Therefore, a multifunctional role of each of these secretions might be dependent on the context of the TME, including different cytokine and chemokine secretions.

MDSCs are attracted to infiltrate CRC and other solid tumors via production of different cytokines, including IL-17, IL-8, IL-6, TNF-α, and GM-CSF (Chen et al., 2014; Wu et al., 2014). IL-6 is considered one of the important cytokines in MDSC infiltration and regulation, as indicated in a study that showed that IL-6 inhibition in tumor-bearing mice depletes MDSCs and increases IFN-γ production by CD4+ and CD8+ T cells (Sumida et al., 2012). It should also be noted that MDSCs communicate with Tregs and can stimulate their development *in vitro* and *in vivo* via IL-10 and TGF-β (Huang et al., 2006). IL-6 is one of the abundant secretions of SASP as mentioned above besides the other inflammatory mediators.

Immune checkpoints are vital factors mediating immune evasion in the TME. CRC cells secrete several immune checkpoint molecules that are associated with T cell inactivation, including PD-L1, CTLA-4, and LAG-3 (Llosa et al., 2015). Mismatch repair deficiency (MMR-D) microsatellite instability high (MSI-H) colorectal carcinomas are characterized by high lymphocyte infiltration (Smyrk et al., 2001). These types of cancers are also characterized by a large number of mutations that are associated with neoantigens (Segal et al., 2008; Fabrizio et al., 2018; Sahin et al., 2019) that can be recognized by infiltrating T cells (Dolcetti et al., 1999). Patients who have these types of tumors respond to a PD-1 blockade and exhibit a higher frequency of neoantigen-specific T cell clones (Le et al., 2017). While MSI colorectal cancers are characterized by high infiltration of immune cells (CTL and Th1 cells), they also express high levels of several immune checkpoint molecules (Llosa et al., 2015; Fabrizio et al., 2018; Sahin et al., 2019). These immune checkpoints, such as PD-L1, block the activation of the immune cells against the tumors and CD8 T cell proliferation is less when they are close or in contact with PD-L1-expressing tumor cells (Le et al., 2015; Marisa et al., 2017). The anti-tumor immune cell activity interrupted by PD-L1 is suggested to be mediated by activation of the Akt-mTOR signaling pathway (Lastwika et al., 2016). This signaling pathway and PD-L1 expression have been shown to be stimulated in prostate cancer cells via the senescent stromal SASP AREG molecule (Xu et al., 2019).

Since senescence has been associated with inflammatory secretions, it could contribute in mediating hot or inflamed tumors, characteristic of MSI tumors (Yoshioka and Matsuno, 2020). These tumors show a better response to immunotherapy, particularly immune checkpoint blockade. Turning cold tumors to hot tumors by senescence induction combined with immunotherapy could be a promising therapeutic approach (Duan et al., 2020). CD95L displays a correlation with MSI colon cancer (Raats et al., 2017). CD95L induced senescence in MSI cancer cells is indicated by SA-β-gal upregulation, reduction in Ki67 and other senescent markers. CD95L also induces SASP secretions particularly inflammatory molecules (Raats et al., 2017). On the other hand, microsatellite stable (MSS) tumors have fewer neoantigens, lower immune checkpoints expression and immune infiltration, and acquire resistance to immune checkpoint inhibitors (Sahin et al., 2019). In contrast to the above studies suggesting a correlation between senescence and MSI tumors, a recent study has reported that MSS CRC tissues displaying senescent epithelial cell accumulation are associated with low immune cell infiltration (Choi et al., 2021). For instance, p16 positive tissues showed a low density of intratumoral CD8 T cells infiltration, whereas p16 negative tissues showed higher CD8 T cells infiltration. Senescent cells secreted CXCL12 inhibiting CD8 T cell infiltration and CSF1 induced differentiation of monocytes into M2 macrophages (Choi et al., 2021). In addition, inhibiting these two SASP molecules enhanced the effect of an immune checkpoint inhibitor (anti-PD-1) in allograft tumors (Choi et al., 2021). Therefore, it could be better for future studies to compare MSI with MSS in terms of their response to senescence inducers as well as immune scoring in tumor tissues from patients treated with a chemotherapy that is known to induce senescence.

#### A Role for ILK in Senescent Cells and Immune Cells

ILK may play a role in cellular senescence in different contexts including aging and cancer. It has been reported that ILK is upregulated in tubular epithelial cells and fibroblasts isolated from old rats compared with young rats, with expression positively correlated with SA-β-gal (Li et al., 2004; Chen et al., 2006). Knockdown of ILK expression by siRNA in rat cardiac fibroblasts prevented the phenotypes of senescence (Chen et al., 2006). Also, overexpressing ILK in young and old rat cardiac fibroblasts induced SA-β-gal (Chen et al., 2006). However, in the cancer context, it is suggested that ILK may display an opposite function. For example, cellular senescence markers like SA-β-gal are induced in skin tumors and benign colon adenomas by peroxisome proliferator-activated receptor-β/δ (PPARβ/δ) via repressing ILK and pAkt (Zhu et al., 2014). Also, in different cancer models such as retinoblastoma and glioblastoma cell lines, ILK inhibition by either QLT-0267 or ILK RNA interference increases SA-β-gal in Rb-positive cells (Duminuco et al., 2015). These studies are suggesting a regulatory function of ILK in cellular senescence and that appears to be context dependent. However, it has not been investigated whether ILK is directly linked to senescent cancer cells or not. Also, the SASP secretions, the hallmark of deleterious senescence, are absent.

SASP secretion is critical in the function of senescence, and ILK or its downstream signaling partners including PTEN, PI3K/Akt/mTOR, and NF-κB may have an effect on SASPs regulation. PTEN is a negative regulator of ILK and Akt, and its deficiency leads to activation of ILK and Akt (Persad et al., 2000; Hähnel et al., 2008). It has been reported that loss of PTEN induced cellular senescence in prostate cancer in mice (Toso et al., 2014). While this was accompanied by immunosuppressive SASP secretions, in contrast, there was also upregulation of immunostimulatory SASPs. This subsequently increased the infiltration of MDSCs, with the absence of CD4 T cells, CD8 T cells, and NK cells (Toso et al., 2014). This study also indicated that SASPs can be programmed by targeting Stat3, resulting in the inhibition of immunosuppressive secretions while maintaining immunostimulatory secretions. Consequently, this led to a decrease in infiltrating MDSCs and an increase of CD4 T cells, CD8 T cells, B cells, and NK cells (Toso et al., 2014). As NF-κB is a master regulator for SASP activation, inhibiting it blocks SASP and sensitizes cancer cells to chemotherapy (Musiani et al., 2020). Our laboratory has reported that ILK inhibition in a mouse model of colitis blocked NF-κB (IKK and p65) activation, and suppressed TNF-α, IL-6, and IL-1β production as well as infiltration of inflammatory cells (Ahmed et al., 2014, 2017). Therefore, ILK may have an impact on SASP secretions in the cancer context, possibly by regulating NF-κB.

The production of SASP obviously has a link with immune cell recruitment and consequently it is essential to consider the role of ILK and its partners in immune cell activation. Deleting PTEN in CRC HCT116 cells leads to constant Akt activation, which in turn increases cell resistance to cytotoxic T cells *in vitro*, as well as to adoptively transferred murine splenocytes *in vivo* (Hähnel et al., 2008). Moreover, pharmacological inhibition of Akt in cultured melanoma-infiltrated lymphocytes enables their expansion with transcriptional signature of memory T cells (Crompton et al., 2015). Similarly, flow cytometry analyses of cells harvested from mice spleens show that PI3K-Akt inhibition does not affect the total number of CD4 T cells in comparison with non-treated mice, whereas the balance between Foxp3+ Treg cells and CD8+ T cells is altered by increasing CD8+ T cells and decreasing Foxp3+ Treg cells (Abu-Eid et al., 2014). When Akt is inhibited that augments the T cell response to peptide vaccination by IFN-γ secretion (Abu-Eid et al., 2014). Furthermore, ILK and MMP have a clear link (Troussard et al., 2000), and MMP is one of SASP molecules that has a role in immune evasion in senescent cells by shedding NKG2D ligands to avoid NK cell killing (Muñoz et al., 2019). The above reports suggest that ILK could be implicated in regulating immune cell function in a cancer context.

ILK may also have a role in the expression of immune checkpoint, which as discussed above is a barrier to anti-tumor immunity. Different lung cancer cell lines and the CRC HCT116 cell line express high levels of PD-L1, mediated by activation of the Akt-mTOR pathway *in vitro*, in accord with a finding *in vivo* in murine lung tumors (Lastwika et al., 2016). Similarly, high activation of the PI3K/Akt/mTOR pathway in colon cancer stem cells prepared from HT-29 spheroids is induced by insulin and this is accompanied by high expression of PD-L1 (Chen et al., 2019). A more recent study has revealed that glioblastoma patients that lack a response to anti-PD-1 have high PTEN mutations and Akt activation in comparison to patients showing a better response (Zhao et al., 2019). A combination of PD-1 blockade with Akt-mTOR inhibition reduced the tumor growth, accompanied by a decrease in Foxp+ Treg cells and an increase in CD3+ T cells, indicating immune activation (Zhao et al., 2019). In addition to immunity enhancement, apoptosis and senescence are induced by such a combination therapy (Lastwika et al., 2016).

Taken together, the above studies indicate that ILK could have an impact on regulating cellular senescence and SASP secretion which has been established to be connected to immune cell recruitment and activation in the TME. This regulation could be mediated directly by ILK or indirectly via ILK partners pathway such as PTEN, PI3K, Akt, mTOR, and NF-κB. Targeting ILK could be an option to induce senescence and regulate the inflammatory response in tumors. Moreover, activating immune cells combined with targeting ILK also should be taken in consideration to maintain and augment immune responses against tumors.

### TLR Stimulation Combined With Targeting ILK in Cancer Context

Activating tumor-specific immunity has been a goal for decades, invoking both the innate and adaptive immune systems. More recently, since the description of TLRs and their agonists, new strategies have been devised for targeting different cancers (Li et al., 2014; Javaid and Choi, 2020). TLRs are a class of pattern recognition receptors that have the capacity to recognize and bind specific molecules (pathogen-associated molecular patterns or PAMPS) released by pathogens, including bacteria and viruses. They can also bind endogenous molecules (danger-associated molecular patterns or DAMPs) released from stressed or damaged cells (Apetoh et al., 2007; Chen and Nuñez, 2010). PAMPs and DAMPS bind and activate TLRs to trigger innate immune responses and subsequently to prime adaptive cellular immunity (Hornung et al., 2002; Iwasaki and Medzhitov, 2004). While TLRs are expressed by different innate immune cells including DCs and macrophages, other cells from both the innate and adaptive system, as well as fibroblasts and epithelial cells, can respond to TLR agonists (So and Ouchi, 2010; Dajon et al., 2017). Interestingly, TLRs are also expressed in many tumors and their role is context dependent and may be positive or negative, by either suppressing or promoting cancer progression, respectively (So and Ouchi, 2010).

Depending on ligand type, TLRs are located on either the cell membrane (e.g., TLR1, 2, 4, 5, and 6) or on endosomal membranes (e.g., TLR3, 7, 8, and 9). The cell membrane TLRs bind proteins or lipids, whereas endosomal membrane TLRs bind nucleic acids (Rakoff-Nahoum and Medzhitov, 2009). TLR signaling is initiated by ligand binding and signal transduction through adaptor proteins, myeloid differentiation primary response-88 (MyD88) and TIR-domain-containing adapter-inducing interferon-β (TRIF) (Wang et al., 2018). All TLRs activate MyD88 except TLR3, which interacts with TRIF. Also, IL-1 receptor families signal via MyD88 (Wang et al., 2018). These adaptors activate transcriptional factors NF-κB, activator protein 1 (AP-1) and interferon regulatory factor 3 (IRF-3) (Piras and Selvarajoo, 2014), mediating gene expression of cytokines including TNF-α, IL-1β, IL-6, interferon gamma-induced protein 10 (IP-10) and IFN-γ (Piras and Selvarajoo, 2014).

As reviewed recently, stimulating TLRs combined with chemotherapeutic agents, which are known induce senescence, may suppress tumor growth and stimulate anti-tumor immunity (Apetoh et al., 2007; Urban-Wojciuk et al., 2019). The TLR3 agonist poly I:C efficiently suppressed the growth of CT26 colon tumors in mice and induced anti-tumor NK cells (Takemura et al., 2015). Furthermore, another study has reported that TLR3, 4, and 7 expression in primary CRC cells and immune cells enabled targeting with combined agonists, activating immune-cell- directed killing of CRC cells in coculturing assays (Stier et al., 2013). Also, *in vivo* the TLR agonists suppressed tumor growth and the effect was improved by using a combination of the agonists or together with chemotherapy (Stier et al., 2013). Our laboratory has previously shown that combination of TLR agonists is synergistically active against the C57Bl6 melanoma model in mice (Whitmore et al., 2004).

In contrast, TLR activation induces inflammation and this is implicated in carcinogenesis and cancer progression (Terzić et al., 2010). The nature of TLR agonists and tumor type have to be taken into consideration for designing cancer therapeutics (Dajon et al., 2017). Damaged or senescent cells could release DAMPS to activate TLRs activation (Hari et al., 2019), then TLR activation such as TLR-2 may have a suppressive effect on tumor growth (D’Agostini et al., 2005). However, this TLR has been found to mediate SASP induction via activating a master regulator of SASP, NF-κB (Piras and Selvarajoo, 2014). Subsequently, this suggests that combining TLR stimulation with targeting particular proteins implicated in cancer progression and inflammation should also be considered. Targeting ILK may present a therapeutic option in this regard. Since ILK mediates activation of the TLR4/NF-κB/TNF-α signaling pathway by LPS (a TLR4 agonist) in colitis (Ahmed et al., 2014), it is possible that ILK could have an impact on regulating the activities of different TLRs in cancer. Therefore, targeting ILK combined with TLRs stimulation and senescence induction could be a potentially synergetic approach to regulate senescence positively, stimulate innate immunity and ultimately suppress tumor growth.

## Conclusion

**FIGURE 5:**
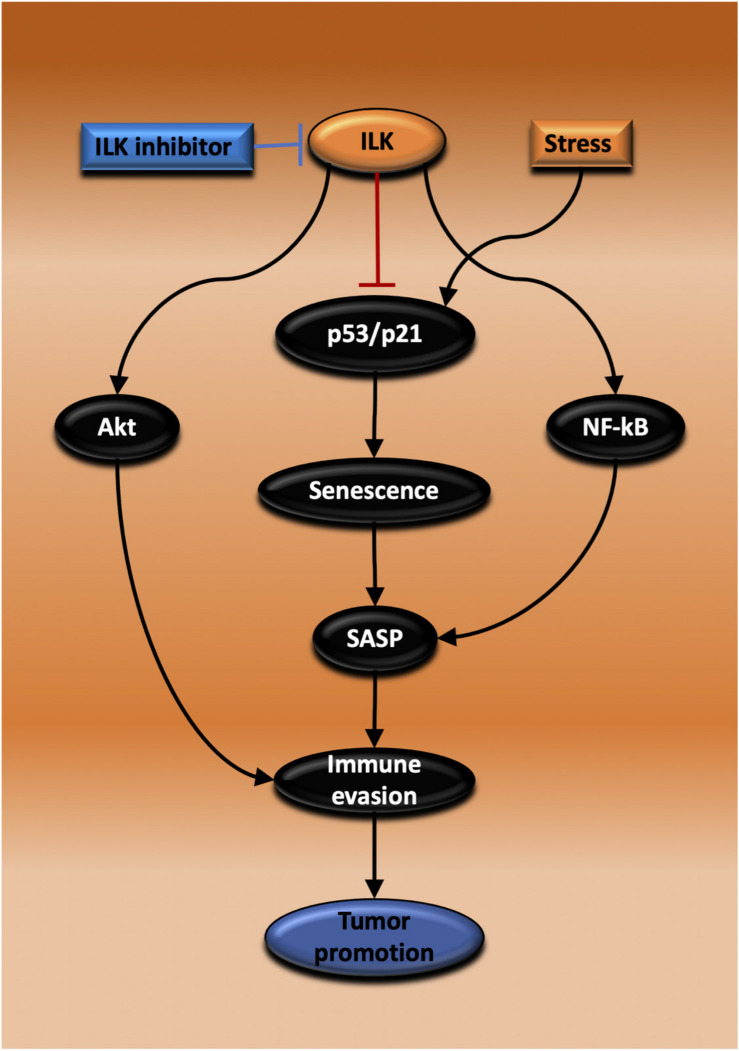
Proposed mechanisms for the promotion of tumor growth by ILK via regulation of senescence and immunity. Inhibiting ILK could suppress and regress CRC growth by three possible mechanisms that are dependent on each other. In the first, ILK inhibition suppresses the activation of Akt, maintaining immune cell activation and tumor cell killing. In the second mechanism, ILK inhibition increases senescence induction via activating p53/p21, thereby inhibiting tumor growth. The third possibility relies on ILK inhibition and the consequent suppression of NF-κB activation, a master regulator of SASP, reprogramming SASP secretion to induce immune surveillance.

Chronic inflammation as evidenced by IBD is a risk factor initiating CAC or CRC. Inflammatory response recruits inflammatory cell infiltration and that leads to tumor initiation. Inflammation is also involved in all stages of cancer including growth, invasion and metastasis. Chemotherapy or radiotherapy treatment of tumors may prevent growth temporarily; however, after a period, the tumors will regrow. Different bodies of evidence show that arresting tumor growth is mediated by TIS. However, because the induced senescent cells are still active in metabolism and transcription, they will produce SASPs. There are pronounced heterogeneous inflammatory molecules in SASP, the SIR, that have an impact on rescuing tumor growth again and promote progression via different mechanisms including growth arrest escape, immunosuppressive cell infiltration and immune evasion. ILK has been implicated as a mediator in both inflammation and tumor growth. ILK also has been suggested to prevent senescence induction in the cancer context. Nonetheless, the knowledge about the role of ILK in cellular senescence and inflammatory response mediated by SASPs remains incomplete. Targeting ILK in solid tumors such as CRC could be effective in suppressing tumor growth via promoting TIS, enabling recruitment of anti-tumor immune cells into the TME thereby regulating SASP secretion (Figure 5). What is necessary is further understanding of the function of ILK in epithelial and inflammatory cells during a senescence induction and investigation of the inflammatory senescence secretions and their effect on tumor growth. Also, it is important to examine the role of ILK in immune cell infiltration recruited following senescence induction. Finally, TLR stimulation could be an effective therapy combined with ILK inhibition and TIS for triggering cytotoxic immunity and regressing tumor growth.

## Author Contributions

SA and BW planned the review. SA drafted. AA, RB, and BW edited the review. All authors contributed to the article and approved the submitted version.

## Conflict of Interest

RB was Chief Scientific Officer and shareholder of Catherics Pty Ltd. BW was Director and shareholder of this company. The remaining authors declare that the research was conducted in the absence of any commercial or financial relationships that could be construed as a potential conflict of interest.
